# Habitat selection by free-roaming domestic dogs in rabies endemic countries in rural and urban settings

**DOI:** 10.1038/s41598-022-25038-z

**Published:** 2022-12-03

**Authors:** Laura Cunha Silva, Brian Friker, Charlotte Warembourg, Kaushi Kanankege, Ewaldus Wera, Monica Berger-González, Danilo Alvarez, Salome Dürr

**Affiliations:** 1 Veterinary Public Health Institute, Vetsuisse Faculty, University of Bern, Bern, Switzerland; 2 Graduate School for Cellular and Biomedical Sciences, University of Bern, Bern, Switzerland; 3 College of Veterinary Medicine, University of Minnesota, St Paul, MN USA; 4 Kupang State Agricultural Polytechnic (Politeknik Pertanian Negeri Kupang), West Timor, Indonesia; 5 Universidad del Valle de Guatemala, Guatemala City, Guatemala; 6 Swiss Tropical and Public Health Institute, Basel, Switzerland

**Keywords:** Behavioural ecology, Ecological epidemiology, Urban ecology, Ecology

## Abstract

Domestic dogs can affect human health through bites and pathogen transmission, particularly in resource-poor countries where dogs, including owned ones, predominantly roam freely. Habitat and resource selection analysis methods are commonplace in wildlife studies but have not been used to investigate the environmental resource use of free-roaming domestic dogs (FRDD). The present study implements GPS devices to investigate habitat selection by FRDD from an urban site and a rural site in Indonesia, and one urban and two rural sites in Guatemala (N = 321 dogs). Spatial mixed effects logistic regression models, accounting for heterogeneous distribution of the resources, showed that patterns of habitat selection by FRDD were similar across study sites. The most preferred resources were anthropogenic, being buildings and roads, which implies selection for human proximity. Vegetation and open fields were less preferred and steep terrain was avoided, indicating that FRDD were synanthropic and that their space patterns likely optimised energy use. Results presented here provide novel data on FRDD habitat selection patterns, while improving our understanding of dog roaming behaviour. These findings provide insights into possible high-risk locations for pathogen transmission for diseases such as rabies, and can assist management authorities in the planning and deployment of efficient disease control campaigns, including oral vaccination.

## Introduction

Free-roaming domestic dogs (FRDD, *Canis familiaris*) are owned or unowned dogs allowed to roam freely and commensally under no direct human supervision, and which retain dependence on humans^1^. Most domestic dogs in low and middle-income countries are free-roaming due to traditional dog management practices, fast urban expansion, and failure to prioritise dog population control^2^. Negative impacts of FRDD on public health have been thoroughly studied^3–6^, including their role in the transmission of pathogens causing zoonotic diseases^6^. Rabies is the dog-transmitted zoonotic disease with the highest burden to human health^7^. An estimated 99% of human rabies cases worldwide are caused by dogs, particularly FRDD, occurring in low- and middle-income countries^8,9^.

Reducing knowledge gaps on dog ecology is required to improve disease control strategies, which previous literature has explicitly indicated as necessary for improving rabies control^8,10^. Consequently, investigations on dog roaming behaviour have become more prevalent^11–16^. Domestic dogs have a unique ecology because, although they are a domesticated species mostly dependent on humans, when unrestrained and allowed to roam freely, they also exhibit behaviours that are common to other wild-living canids such as large range roaming, hunting scavenging, and territory defence^17^. Investigations on how FRDD use their anthropogenic and natural environments is therefore of interest to further understand their roaming behaviour. Habitat selection methods have often been used in wildlife studies, including research on carnivores^18–20^. Such methods have not been applied to investigate how FRDD use different resources in habitats that they occupy. A habitat is defined as the environment that animals use to survive and to proliferate^21^, and is comprised of a combination of biotic and abiotic resources that impact the presence or absence of an organism^22^. The selection of these resources by an organism for obtaining their needs is defined as habitat selection^22,23^.

Knowledge about FRDD habitat selection is necessary to identify relevant locations and environmental resources that are high risk for pathogen transmission and to guide responses for disease outbreaks or endemism^24^. As previously done with other species and viruses^25^, determining FRDD habitat preferences could improve the understanding of the spatial distribution related to the risk of zoonotic disease transmission. Thus, identifying resources that are disproportionately chosen or avoided by FRDD across different locations, can guide policy makers in strategic allocation of funding for optimal disease prevention and control. For example, knowledge generated from the present study is relevant for informing strategies related to oral rabies vaccination campaigns, which has been discussed as an essential method to achieve high vaccination coverage in poorly accessible FRDD and feral dog populations^7,26–28^.

By analysing data on FRDD in five distinct settings in two different low- to middle-income countries, we investigated resources meaningful to FRDD habitat selection in relation to anthropogenic and geographical characteristics in rural and urban landscapes. In particular, we aimed at evaluating whether FRDD specific habitat selection differed across sites and regions.

## Results

One hundred and 52 FRDD were equipped with a geo-referenced contact sensor (GCS) in Habi and Pogon, Indonesia, respectively. In Poptún, 118 dogs were equipped with a GCS device, 61 in La Romana and 125 in Sabaneta. In Habi and Pogon, Indonesia, respectively, 73 and 36 GCS devices were viable for the analysis. Reasons for unviable GCS devices in Habi were due to malfunctions related to recording GPS fixes, one device was lost, three damaged and four did not record any data. In Pogon, no devices were lost but two were damaged. In Guatemala, data from 69 GCS devices in Poptún, 98 in Sabaneta and 45 in La Romana were viable for the analysis. In Poptún, besides devices that had not recorded GPS fixes, four devices were lost and four were damaged. In Sabaneta, two devices were lost and four damaged, and in La Romana, three devices were lost and six were damaged. This resulted in a total of 321 FRDD included in our study, ranging from 36 to 98 per study site (Table 1).

**Table 1:** Resource coverage and steepness of the terrain of the study sites to investigate habitat selection of 321 free-roaming domestic dogs (FRDD) in Guatemala and Indonesia.

	Habi (semi-urban)	Pogon (rural)	Poptún (urban)	La Romana (rural)	Sabaneta (rural)
**Country**	Indonesia	Indonesia	Guatemala	Guatemala	Guatemala
**Number of FRDD from which GPS data was analysed**	73	36	69	45	98
**Resource area**					
Roads (% coverage)	164,277 m^2^ (3.1%)	26,788 m^2^ (0.7%)	731,862m^2^ (13.1%)	32,557 m^2^ (0.3%)	80,783 m^2^ (0.2%)
Buildings (% coverage)	503,549 m^2^ (9.4%)	59,706 m^2^ (1.6%)	1,582,569 m^2^ (28.9%)	40,005 m^2^ (0.4%)	129,739 m^2^ (0.3%)
Low vegetation (% coverage)	323,008 m^2^ (6.0%)		3,253,546 m^2^ (58.4%)	7,635,768 m^2^ (75.9%)	43,729,185 m^2^ (99.5%)
High vegetation (% coverage)		3,639,390 m^2^ (97.7%)		2,356,859 m^2^ (23.4%)	
Beach (% coverage)	17,566 m^2^ (0.3%)				
Sea (% coverage)	215,534 m^2^ (4.0%)				
Open field (% coverage)	4,124,915 m^2^ (77.1%)				
**Range of the terrain slope in degrees**	− 1.8^a^ to 1.8	1.7–50.8	0–18.6	0–48.3	0–50.2
**Range (median) of slope in degrees of the observed GPS fixes**		2.0–51.0 (17.0)	0.2–11.0 (2.4)	0.7–38.0 (4.0)	0.0–39.8 (2.7)

Building-like structures were identified in all study sites making up the “buildings” resource. “Roads” were also identified in all sites. Sparse vegetation and bushes present in all sites except Pogon were labeled as “low vegetation”, and dense forest-like vegetation present in La Romana and Pogon as “high vegetation”. In Habi, aside from the aforementioned resources, “beach”, “sea” and “open fields” were also identified.

^a^Available habitat includes areas below sea level.

The study population in Indonesia consisted of young dogs (median recorded age in Habi: 10 months; median recorded age in Pogon: 12 months) with the majority being females (61%). The majority of dogs in Indonesia were entire (85% in Habi and 94% in Pogon). In Indonesia, all dogs were reported to serve as guardians, some of them were also declared as pets (19%) or as meat sources (10%). In Guatemala, the FDRR study population consisted mainly of males (67%). Almost all dogs included in our study in Guatemala were entire (100% in Poptún, 96% in Sabaneta and 89% in La Romana). Dog ages were not consistently recorded in Guatemala. Dogs were mainly kept as guardians (52%), and in contrast to Indonesia, they were not considered meat sources.

Resource coverage differed across the study sites. In Habi, "open field" was the resource with the highest coverage percentage (77.1%). In Pogon "High vegetation" covered 97.7% of the available habitat. In all Guatemalan sites "Low vegetation" was the resource with the highest coverage percentage (Poptún: 58.4%; La Romana: 75.9%; Sabaneta: 99.5%). The terrain was rather steep in Pogon, La Romana and Sabaneta, ranging up to 50 degree for Pogon and Sabaneta, whereas only moderately steep in Poptún (Table 1). The study site area in Habi was found to be extremely flat.

Similarities in habitat selection were identified between study sites. Here we present the results with “roads” as the given reference level (Table 2). Supplementary tables included in the appendix implement other reference levels. In Habi, La Romana and Sabaneta, FRDD preferred residing near “buildings”, while in Pogon and Poptún, “roads” were the most selected habitat resource (Table 2). In Habi, a semi-urban area of Indonesia, dogs were less often present in “beach”, “low vegetation”, “open fields” and “sea” compared to “roads” and “buildings”, with differences being statistically significant for all but “beach”. “Low vegetation” was significantly preferred over “open fields” (OR = 1.83) (Supplementary Table 1). In Pogon, a rural area in Indonesia, FRDD were less likely present in “buildings” (OR = 0.78) and “high vegetation” (OR = 0.08) compared to “roads”, with significant differences for “high vegetation” only. In Poptún, an urban site in Guatemala, “buildings” (OR = 0.87) and “low vegetation” (OR = 0.26) were found to be significantly less preferential compared to “roads”, and “low vegetation” were less preferred than “buildings” (OR = 0.30) (Supplementary Table 2). In La Romana, a rural area in Guatemala, dogs were found to be significantly more present in “buildings” (OR = 3.17), but significantly less present in both “high” and “low vegetation” areas (OR = 0.02; OR = 0.12, respectively) with reference to “roads”. When it comes to vegetation preferences, dogs showed less preference for “high vegetation” compared to “low vegetation” (OR = 0.15) (Supplementary Table 3). In Sabaneta, the other included rural area in Guatemala, “low vegetation” was significantly less preferred (OR = 0.06), and “buildings” were significantly more preferred (OR = 2.35) compared to “roads”.

**Table 2:** Results (odds ratio and 95% confidence interval for the explanatory variables habitat, time and slope) of the spatial mixed effects logistic regression model with “roads” as the resource reference level of 321 free-roaming domestic dogs in Indonesia and Guatemala.

	Number of observed GPS fixes	Number of randomly generated GPS fixes	Odds ratio (OR)	95% confidence interval of OR
**Habi–Indonesia**				
**Habitat resources**				
Roads	2560	2827	Reference level	
Buildings	51,328	8480	8.13	7.65–8.64
Low vegetation	4982	5707	0.83	0.77–0.86
Beach	227	268	0.90	0.74–1.09
Open fields	28,548	67,087	0.45	0.43–0.48
Sea	255	3531	0.08	0.07–0.09
Hour			0.99	0.99–0.99
**Pogon–Indonesia**				
**Habitat resources**				
Roads	1176	78	Reference level	
Buildings	2490	225	0.78	0.59–1.02
High vegetation	9593	12,956	0.08	0.07–0.11
Slope			0.88	0.88–0.89
Hour			0.98	0.97–0.98
**Poptún–Guatemala**				
**Habitat resources**				
Roads	9451	5034	Reference level	
Buildings	17,491	11,033	0.87	0.84–0.91
Low vegetation	12,215	23,090	0.26	0.25–0.28
Slope			1.02	1.01–1.04
Hour			0.99	0.99–0.99
**La Romana–Guatemala**				
**Habitat resources**				
Roads	1996	186	Reference level	
Buildings	10,134	250	3.17	2.60–3.88
Low vegetation	46,316	46,662	0.12	0.11–0.14
High vegetation	1385	12,733	0.02	0.02–0.02
Slope			0.84	0.84–0.84
Hour			0.99	0.99–0.99
**Sabaneta–Guatemala**				
**Habitat resources**				
Roads	4553	161	Reference level	
Buildings	19,774	258	2.35	1.91–2.91
Low vegetation	66,972	90,880	0.06	0.05–0.07
Slope			0.69	0.68–0.70
Hour			0.99	0.99–1.00

This table details the total number of observed and randomly generated GPS fixes present in each study site resource. Note: Confidence intervals that do not include 1 correspond to a *p*-value of < 0.05 and thus present a significant effect on the α = 0.05.

Although FRDD were found even in steep terrains, slope was negatively correlated with the presence of FRDD in La Romana, Sabaneta and Pogon, with OR ranging from 0.69 to 0.88 for each degree increase in slope, indicating that FRDD preferred flat environments (Table 2). In Poptún, steepness was significantly associated with the presence of dogs, however the effect size with OR = 1.02 was considerably small (Table 2).

## Discussion

This study provides the first evidence of habitat selection of FRDD, a species living close with humans, but still allowed to roam unsupervised partially or entirely. Our findings highlight that “buildings” and “roads”, i.e. man-made structures, are preferred by FRDD, independent of the study location and whether they live in urban or rural settings.

“Buildings” were the most preferred resource in Habi, La Romana and Sabaneta when compared to any other resource type. Human interaction is known to influence dog roaming behaviour^29,30^ and preference to buildings was expected because humans provide dogs with food, water, and shelter. These results concurs with previous findings, mostly from rural settings: for example, in Bulgaria even feral dogs stay close to buildings to have facilitated access to food^31^, whilst in Zimbabwe and India FRDD diets consist mainly of human waste, which dogs retrieved from areas near human buildings^32,33^.

“Roads” were the most used resource of FRDD in Pogon (rural setting) and Poptún (urban setting), compared to all other resources. While Poptún is an urban site, where road density is high with moderate traffic (Fig. 2c), Pogon is a densely forested region, where direct access to buildings is tortuous and road traffic is only sporadic. Roads build pathways for facilitated movement, and often provide access to food sources, such as roadside garbage, collection sites, and possibly animal carcasses^33,34^. Roads influence dog movements and contacts, especially in rural areas^34,35^, a finding also observed in African wild dogs (*Lycaon pictus*)^18,19^ and pumas (*Puma concolor*)^20^. As demonstrated by Sepúlveda et al.^34^, roads also ease movement into the arboraceous areas to foray, highlighting that they may be mainly used as to access any other resource more easily. Dissimilar urban planning and development in the chosen urban study sites may explain the observed difference of the dominant resource (buildings in Habi versus roads in Poptún).

FRDD exhibited preferences for “low vegetation” over "high vegetation", as evidenced in La Romana, the only study site where both vegetation resources could be compared. Foraging activities, which could have been an expected driver for dogs to spend time in the forest, may have been superfluous, since dogs had an easier access to food in areas closest to human presence. In addition, forests may have acted as a barrier to movement^34^. These observations are distinct from feral dog behaviour. A study in Bulgaria found that feral dogs (dogs that, unlike FRDD, are not human-dependent) tend to prefer dense vegetation covered areas^31^. Comparisons with wildlife species demonstrated differences between pumas in Mexico that preferred zones with higher tree coverage (woodland)^20^, while African wild dogs in Kenya preferred less tree coverage^18^, reflecting the vegetation structure of their habitats. In Habi, “low vegetation” was significantly preferred over “open fields”, which, in these hot climate areas, could be because trees offer dogs shade, protecting them from the sun.

The “sea” resource in Habi was less used than most of the other resources. Dogs may be capable of swimming, but such activity is rare, according to our personal observations and GPS recordings in Habi. However, misclassification of GPS fixes between beach and sea cannot be excluded since satellite imagery, a snapshot of the landscape at a given time, is conditional to the time the image was captured and influenced by sea tides and terrain conditions. Nevertheless, it can be concluded that both, “beach” and “sea” are resources that are visited by dogs occasionally, but do not constitute an essential habitat, an observation that is in line with previous research in far northern Australia^35^.

Flat slopes were favored by FRDD in all sites, likely due to the ease of movement and foraging behaviour in flat areas. Compared to other wildlife species, slope preference has been investigated in prairie dogs^36^ and pumas^20^, where the same evidence of predilection for flat slopes was reported. These findings infer that topographic features e.g. steep slopes, such as hills, mountains, and volcanos, can serve as hurdles for animal movements, which may be relevant for strategic infectious disease control. In our study, we could demonstrate that even within a relatively small area, steep structures are avoided and may be used as natural barriers to support disease control interventions. Poptún was the only site where FRDD did not significantly avoid steeper locations. With an OR very close to 1 (1.02) the effect is nearly absent, despite the large number of observations that yielded to a significant result. Although Poptún's topography presents some slant (0—18.6 degrees), steepness of the terrain is much lower than for the other study sites, which may explain why it was not shown to have a large effect on FRDD abundance.

Our FRDD study population consisted of dogs allowed to persistently roam freely and those only allowed to roam during parts of the day. Such fact could lead to result bias, as dogs that are only allowed to roam for certain periods of time would produce an excess of GPS fixes in “buildings”, without it reflecting the dog’s own resource choice. Additionally, we did not record the exact times of confinement, which makes it impossible to exclude those periods of confinement from the dataset. Nevertheless, the model’s random effect being defined as each dog’s household, accounts for such a possible result bias.

Another limitation of this study is the cross-sectional design with one single observation period. We were therefore not able to investigate the effect of the season on habitat selection. In addition, this study relies on a relatively short duration of the collaring period. Long-term collaring would have provided a more detailed and reliable insight on dog’s movements. Although shortly discussed in previous studies^37^, the minimal required observation period for FRDD to capture a representative roaming behavior is not systematically studied yet. However, given our large dataset and clear results, we do not believe that the general findings concerning the preference of anthropogenic resources (buildings and roads) would be refuted by these limitations.

Since our primary interest was to draw population-level inferences, the within-individual autocorrelation was overlooked in the context of the analysis ^38^. The statistical analysis used in the present study assumes no clustering of dogs outside from their homes, which cannot be fully accepted for FRDD living in closed neighbourhoods. Therefore, we are not able to draw conclusions on resource use of each individual dog, but only on population level.

Data loss, data precision, and malfunctioning are frequent liabilities when opting for GPS technology^39^. In areas with more interference (i.e. with large trees or between high buildings), satellite signal caption is more challenging^40^. Considering that recording success was reduced in and around high buildings and in high vegetation areas, compared to the other resource areas investigated in this study, this limitation would only influence the findings in Poptún (the only location where high buildings were found) and Pogon and La Romana (where high vegetation was present). Since the other areas are not affected, we believe that the overall conclusion of the study findings would remain the same. In addition, GPS fix records are never fully accurate. It may therefore be that the GPS fixes recorded in one resource was generated from a dog that was actually located in another resource. However, since we have no evidence of GPS accuracy largely differing between resources, we can expect non-biased GPS errors across the study sites, balancing out the inaccurate positions between resources.

Findings of this study provide new knowledge of FRDD roaming behaviour, which can be used to help develop control programs to minimise spread of infectious diseases, such as canine rabies. Low vaccination coverage for rabies in dogs still exists in resource-poor countries, and improving it is the key strategy to effectively eliminate the disease from dog populations^41^. The inaccessibility for parental vaccination of some dogs, such as poorly managed FRDD, is one challenge that leads to low vaccination coverage^7^, and oral rabies vaccination can thus be a complementary tool for them. Oral rabies vaccination is a complementary tool to parenteral vaccination that allows access to otherwise inaccessible dogs. The oral rabies vaccination has been successfully administered for the elimination of rabies in wildlife, and has also shown numerous benefits in studies with FRDD^7,28^. Oral rabies vaccination strategies can be improved by ensuring adequate oral bait distribution in locations known to be visited by free-roaming dogs^26^. Our study supports bait distribution in and around human made resources, including buildings and roads, where FRDD spend more of their time. Combined with the information on temporal activity peaks of dogs in these regions^38^, time and space of an efficient bait distribution can be guided.

## Methods

### Study sites and study design

The study was performed in the frame of a dog ecology research project, with details on the study locations published elsewhere^15,42,43^. For the current study, five study sites located in Indonesia and Guatemala were included. Site selection was carried out by each country’s research team, taking into consideration rural and urban settings, as well as differing expected number of dogs present at each location. The Indonesian study sites were semi-urban Habi and rural Pogon, in the Sikka regency, at the eastern area of Flores Island (Supplementary Fig. 6). In Guatemala, the study sites were Poptún (urban setting), Sabaneta and La Romana (both rural settings), located in the Guatemalan department of Péten, in the northern part of the country (Supplementary Fig. 7). Data were collected during May to June 2018 in Guatemala and from July to September 2018 in Indonesia.

In each location, a 1 km^2^ area was predefined using Google Earth within which the study took place. The 1 km^2^ area was chosen because of the research goals of another part of the project, investigating the contact network of the dogs^15^. Within these areas, the teams visited all dog-owning households. In each household, the study was presented to an adult of the family, who was then asked if they owned a dog and if they were willing to participate in the study. After the dog owner's oral or written consent was granted, a questionnaire was answered, and the dogs collared. The handling of the dogs was performed by a trained veterinarian or a trained veterinary paramedic of the team.

The questionnaire data was collected through interviews with the dog owners. Multiple dogs per household could be included as multiple entries in the questionnaire. The detailed questionnaire contains information on the household location, dog demographics (age, sex, reproductive status) and management (dog’s purpose, origin, confinement, vaccination status, feeding and human-mediated transportation within and outside the pre-determined area).

All dogs of a household fulfilling the inclusion criteria were equipped with a geo-referenced contact sensor (GCS) developed by Bonsai Systems (https://www.bonsai-systems.com), containing a GPS module and an Ultra-High-Frequency (UHF) sensor for contact data recording^43,44^. GCS devices report a 5-m maximum accuracy, a run-time of up to 10 years, can store up to 4 million data points and carry a lithium-polymer-battery (LiPo). For this study, only GPS data were analysed. The GCS were set to record each dog’s geographical position at one-minute intervals. Dogs remained collared for 3 to 5 days with the duration of the data collection being limited by the device’s battery capacity, as batteries were not re-charged or changed during the study. Throughout the time of recording, date, hour, GPS coordinates and signal quality (HDOP) raw data were collected by the GPS module and amassed into the workable databases.

Exclusion criteria were dogs of less than four months of age (since they were not big enough to carry a collar), sick dogs and pregnant bitches (to avoid any risk of stress-induced miscarriages). Reasons for non-participation of eligible dogs included dog owner's absence, dog's absence, inability to catch the dog, and refusal of participation by the dog owner. In addition, dogs foreseen for slaughtering within the following four days were excluded in Indonesia to ensure data collection for at least four to five days. All dogs included in this study were constantly free roaming or at least part-time (day only, night only and for some hours a day). 

Human and/or animal ethical approval were obtained depending on the country-specific regulations. All the procedures were carried out in accordance with relevant guidelines. Ethical clearance was granted in Guatemala by the UVG’s International Animal Care and Use Committee [Protocol No. I-2018(3)] and the Community Development Councils of the two rural sites, which included Maya Q’eqchi’ communities^45^. In Indonesia, the study was approved by the Animal Ethics Commission of the Faculty of Veterinary Medicine, Nusa Cendana University (Protocol KEH/FKH/NPEH/2019/009). In addition, dogs that participated in the study were vaccinated against rabies and/or dewormed to acknowledge the owners for their participation in the study.

### Data cleaning

Data were stored in an application developed by Bonsai Systems compatible with Apple operating system (iOS iPhone Operating Systems), downloaded as individual csv file for each unit, and further analysed in R (version 3.6.1)^46^.

The GPS data were cleaned based on three automatised criteria. First, the speed was calculated between any two consecutive GPS fixes, and fixes with speed of > 20 km/h were excluded, given the implausibility of a dog running at such speed over a one-minute timespan^47^. It is noteworthy that car travel causes speeds over 20 km/h. However, as we were interested in analysing the dog's behaviour outside of car transports, removing these fixes was in line with our objectives. Second, the Horizontal Dilution of Precision (HDOP), which is a measure of accuracy^48^ and automatically recorded by the devices for each GPS fix, was used to exclude fixes with low precision. According to Lewis et al.^49^, GPS fixes with HDOP higher than five were excluded, which deleted 1.3% of data in Habi, 2.2% in Pogon, 3.3% in Poptún, 1.8% in La Romana and 2.1% in Sabaneta. Third, the angles built by three consecutive fixes were calculated for each dog. When studying animals' trajectories as their measure of movement, acute inner angles are often connected to error GPS fixes^50^. The fixes having the 2.5% smallest angles were excluded, to target those fixes with highest risks of being errors, while balancing against the loss of GPS fixes due to the cleaning process. With the exclusion of the smallest angles, 2.6% of data were deleted in Habi, 3% in Pogon, 2.9% in Poptún, 2.6% in La Romana and 2.7% in Sabaneta. After the automatised cleaning was concluded, 18 obvious error GPS fixes (unachievable or inexplicable locations by dogs) still prevailed in the Habi dataset and were manually removed.

### Habitat resource identification and calculation of terrain slope

**Figure 1:**
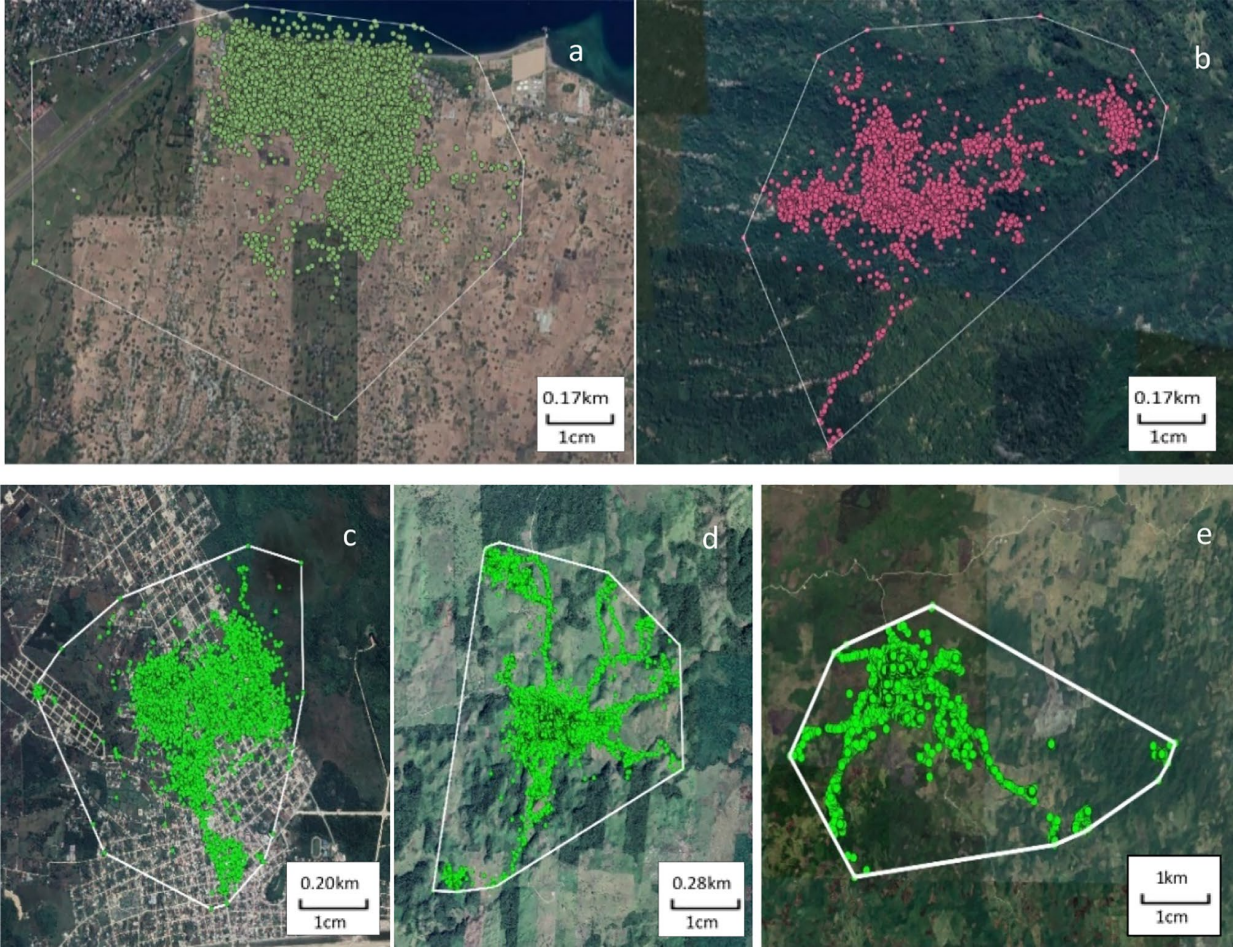
GPS fixes plotted over a Google satellite imagery layer with its respective outlined computed Minimum Convex Polygon (MCP) delimitating the habitat available for the study population in: (**a**) Habi; (**b**) Pogon; (**c**) Poptún; (**d**) La Romana and (**e**) Sabaneta. Source QGIS (version 3.4 Madeira, http://qgis.org), map data: Google Satellite.

To analyse habitat selection of the collared FRDD, resources were delimited by a 100% Minimum Convex Polygon (MCP) including all cleaned GPS fixes per study site, using QGIS^51^ (Fig. 1).

**Figure 2:**
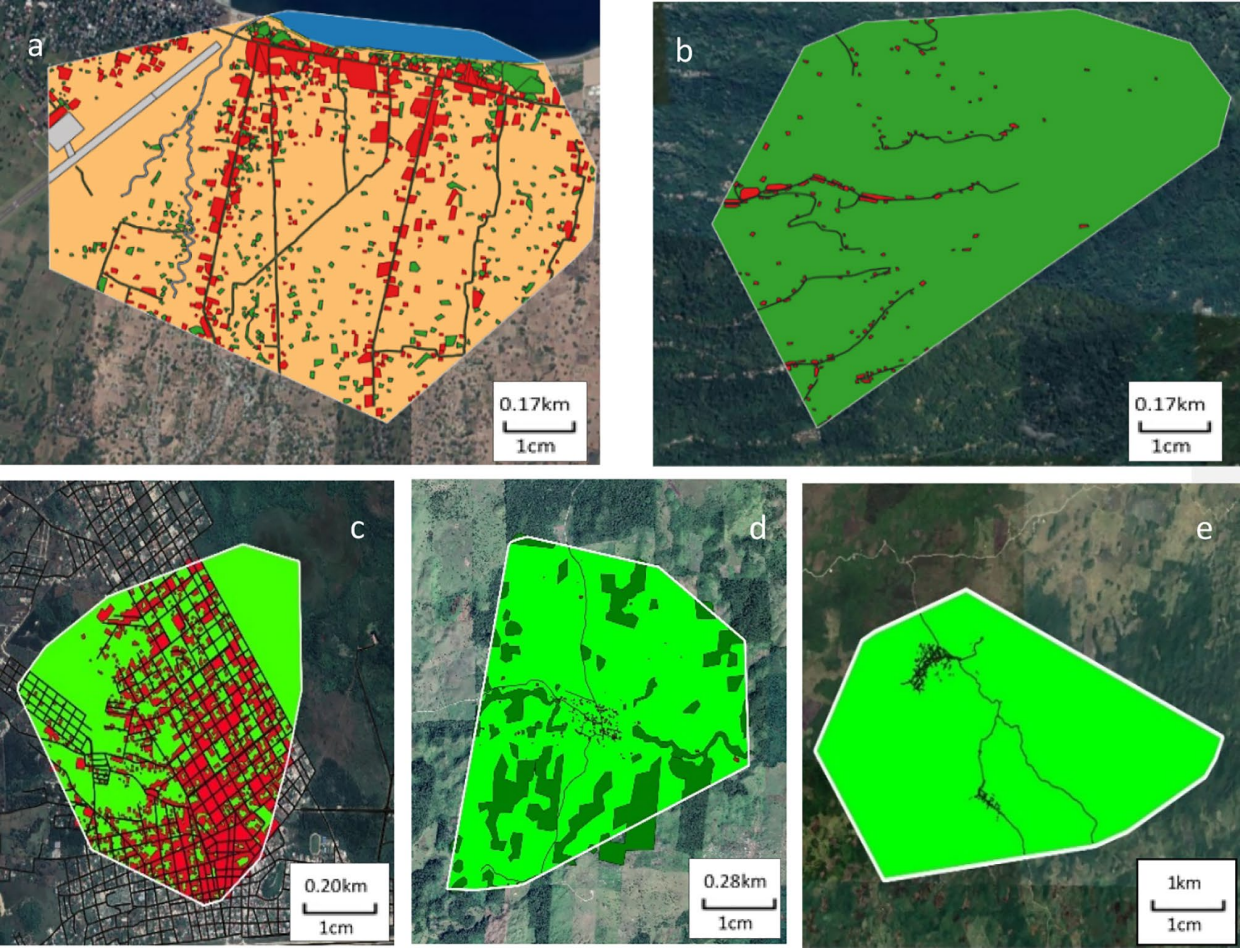
(**a**) Habi, (**b**) Pogon, (**c**) Poptún, (**d**) La Romana and (**e**) Sabaneta Habitat classification vector layers. The different habitat resources, identifiable by colour, were merged to create the comprehensive Habitat classification vector. In the Indonesian sites (**a**, **b**) and Guatemalan sites (**c**–**e**) buildings are coloured red, vegetation low in Habi, Poptún, La Romana and Sabaneta is coloured light green, vegetation high in Pogon and La Romana dark green, roads black, beach yellow, sea dark blue, airport grey, waterways light blue and open field light orange. The airport area (gray) and waterways (light blue) in Habi were not classified as separate habitat layers and were excluded from further analysis. Source QGIS (version 3.4 Madeira, http://qgis.org), map data: Google Satellite.

Resources were defined by taking into consideration the following criteria: resources are (i) likely to impact upon movement patterns of dogs, (ii) identifiable by landscape satellite topography, and (iii) chosen considering information on relevant gathering places for FRDD observed by the field teams. Three resources were disclosed in all study sites: buildings, roads and vegetation coverage. All habitat relevant resources were manually identified within the available area (MCP) in QGIS using satellite imagery. All building-like structures were identified using vector polygons and summed under the layer “buildings”. Roads were identified and manually traced using vector lines in all sites, except in Poptún where the roads were automatically traced using an OpenStreetMap road layer of the area (https://www.openstreetmap.org/export). A buffer vector polygon was generated to encompass the full potential width of the roads, with a 5 m width in Habi and Poptún (semi-urban and urban site) and a 2 m width in Pogon, La Romana and Sabaneta (rural sites). In Habi, a “beach” layer was defined by generating a five-meter buffer from the shoreline in both directions using a vector polygon. The layer “sea” was defined as the vector polygon resulting from the difference between the MCP sea outer limit and the beach buffer polygon. Vegetation coverage was distinct between study sites with sparse vegetation and bushes present in all sites except Pogon, and dense forest-like vegetation present in La Romana and Pogon. These two types of vegetation were defined as “low” and “high vegetation”, respectively. In Habi and La Romana, “low” and “high vegetation”, respectively, were manually identified using vector polygons and summarised under the respective layers. Finally, open field in Habi, high vegetation in Pogon and low vegetation in Poptún, La Romana and Sabaneta were the last vector layers to be established since they represented the difference between all other polygon vector layers and the MCP total area. After all resource vector polygons had been created, an encompassing vector layer was generated by merging all resource polygon vectors for final resource classification (Fig. 2). As part of the resource classification in Habi, the airport terminal and runaway as well as waterways enclosed in the MCP area were identified but excluded from the analysis.

After the construction of the habitat resource layers, all GPS fixes were assigned to the respective resource they were located, using the QGIS *join attributes by location* algorithm. Fixes located exactly on the MCP border in Indonesia were not classified automatically and had to be manually classified to the respective resource.

In non-flat topographies (all locations expect Habi) we tested the hypothesis of whether the steepness would influence the dogs' movement patterns. The degrees of slope were calculated using a 30-m raster-cell resolution (STRM 1-Arc Second Global, downloaded from the United States Geological Survey (USGS) Earth Explorer, https://earthexplorer.usgs.gov/). The slope was assigned by the QGIS *join attributes by location* algorithm to each GPS fix.

### Statistical analysis

To quantify habitat selection in each study site, we compared resources used by the dogs with the resources available, according to Freitas et al.^52^. Adapting the methodology applied by O’Neill et al.^18^, the observed number of GPS fixes for each dog was used to generate an equivalent number of locations that were randomly distributed within the MCP area using the *Random points in layer bound* vector tool from QGIS. For example, if dog “D300” had 100 recorded GPS fixes, 100 random points were generated within the MCP of the respective study site and assigned to “D300”. Random points were then assigned to the respective resources and slope of that location, as previously done with the observed GPS fixes. Using this approach, the habitat resources used by each dog could be compared to the available resources in the respective study site, using a regression model.

Observation independence is a fundamental presupposition of any regression model. However, the spatial nature of the point-referenced data permits perception of spatial dependence. In our dataset, spatial autocorrelation was proven for all study sites using the Moran’s I test. Therefore, we applied a spatial regression model, which takes into consideration spatial autocorrelation while exploring the effects of the study variables. A mixed effects logistic regression model accounting for spatial autocorrelation was created to quantify the effect of variables on used (i.e. observed GPS fix) versus available (i.e. randomly generated GPS fixes) resources, using the *fitme* function in the *spaMM* package in R^53,54^. The model’s binary outcome variable was defined as either observed (1) or random (0) GPS fix, i.e. the dog being present or absent from a position. The explanatory variable was the resource classification with “buildings”, “roads”, “low vegetation”, “beach”, “sea” and “open field” as levels in Habi; “buildings”, “roads” and “high vegetation” in Pogon; “buildings”, “roads”, “low vegetation” in Poptún and Sabaneta; and “buildings”, “roads”, and “high” and “low vegetation” in La Romana. Different habitat resources were used interchangeably as reference level. In all study sites except Habi, the slope was included as an additional explanatory variable. As observations were not evenly distributed in time, with less observations recorded towards the end of the study, a variable ”hour” was added as an additional continuous fixed effect.Each observed GPS fix was assigned to the hour of its record, with the earliest timestamp registered in each study site being assigned the hour zero. The randomly generated points were randomly assigned to an hour within the determined time continuum of the observed GPS fixes. As our focus was investigating habitat selection at a population-level, we assumed there was no within-dog autocorrelation (space/time) and each dog was independent and exhibited no group behaviour^38^. Still, to partially account for spatial autocorrelation of each dog's household, the random effects included in models were defined as each dog’s household geographical location recorded during fieldwork by a GPS device. The restricted maximum likelihood (REML) through Laplace approximations, which can be applied to models with non-Gaussian random effects^55^, and the Matérn correlation function were used to fit the spatial models with the Matérn family dispersion parameter ν*,* indicator of strength of decay in the spatial effect, was set at 0.5^54^.

## Supplementary Information

Supplementary Information 1.

Supplementary Information 2.

Supplementary Information 3.

Supplementary Information 4.

Supplementary Information 5.

Supplementary Information 6.

Supplementary Information 7.

Supplementary Information 8.

Supplementary Information 9.

Supplementary Information 10.

Supplementary Information 11.

## Data Availability

All data required to reproduce the results presented in the manuscript are available within the article and its supplementary material.
