# Supplementary material for: Habitat selection by free-roaming domestic dogs in rabies endemic countries in rural and urban settings
Source: Sci Rep. 2022 Dec 3;12:20928. doi: 10.1038/s41598-022-25038-z (PMC9719531; doi:10.1038/s41598-022-25038-z)
Supplement: Supplementary file 1 — Supplementary Information 1. [file 41598_2022_25038_MOESM1_ESM.docx]

**Habitat Selection by Free-Roaming Domestic Dogs in Rabies Endemic Countries – Rural versus Urban setting**

Laura Cunha Silva^1^*, Brian Friker^1^, Charlotte Warembourg^1^, Kaushi Kanankege^2^, Ewaldus Wera^3^, Monica Berger-González^4,5^, Danilo Alvarez ^4^, Salome Dürr^1^

^1^ Veterinary Public Health Institute, Vetsuisse Faculty, University of Bern, Bern, Switzerland.

^2^ College of Veterinary Medicine, University of Minnesota, St Paul, MN, USA.

^3^ Kupang State Agricultural Polytechnic (Politeknik Pertanian Negeri Kupang), West Timor, Indonesia.

^4^ Universidad del Valle de Guatemala, Guatemala City, Guatemala.

^5^ Swiss Tropical and Public Health Institute, Basel, Switzerland.

**Supplementary Information**

**List of Supplementary Tables**

Supplementary Table 1. – Results for Habi with the open fields resource as reference levels in the spatial mixed effect logistic regression model.

Supplementary Table 2. – Results for all study sites with buildings as reference level in the spatial mixed effect logistic regression model.

Supplementary Table 3. – Results for all study sites with vegetation low resource as reference level in the spatial mixed effect logistic regression model.

Supplementary Table 4. – Results for Habi with the beach as reference level, and for La Romana and Pogon with vegetation high resource as reference levels in the spatial mixed effect logistic regression models for each site.

Supplementary Table 5. – Results for Habi with the sea as reference levels in the spatial mixed effect logistic regression model.

**Supplementary Figures**

Supplementary Figure 1. – Habi's observations resource allocation.

Supplementary Figure 2. – Pogon's observations resource allocation.

Supplementary Figure 3. – Poptún's observations resource allocation.

Supplementary Figure 4. – La Romana's observations resource allocation.

Supplementary Figure 5. – Sabaneta's observations resource allocation.

Supplementary Figure 6: Indonesian study sites localization

Supplementary Figure 7: Guatemalan study sites localization

**Supplementary Table 1.** – Results for Habi with the open fields resource as reference levels in the spatial mixed effect logistic regression model.

|  | **Observed number of observations** | **Randomly generated number of observations** | **Odds ratio (OR)** | **Confidence interval of OR** |
| --- | --- | --- | --- | --- |
| **Habi – Indonesia** |  |  |  |  |
| Habitat |  |  |  |  |
| Roads | 2'560 | 2'827 | 2.21 | 2.07 – 2.32 |
| Buildings | 51'328 | 8'480 | 17.98 | 17.47 – 18.51 |
| Vegetation low | 4'982 | 5'707 | 1.83 | 1.75 – 1.91 |
| Beach | 227 | 268 | 1.99 | 1.66 – 2.39 |
| Open fields | 28'548 | 67'087 | Reference level |  |
| Sea | 255 | 3'531 | 0.17 | 0.15 – 0.19 |
| Hour |  |  | 0.99 | 0.99 – 0.99 |

**Supplementary Table 2.** – Results for all study sites with buildings as reference level in the spatial mixed effect logistic regression model.

|  | **Observed number of observations** | **Randomly generated number of observations** | **Odds ratio (OR)** | **Confidence interval of OR** |
| --- | --- | --- | --- | --- |
| **Habi – Indonesia** |  |  |  |  |
| Habitat |  |  |  |  |
| Roads | 2'560 | 2'827 | 0.12 | 0.12 – 0.13 |
| Buildings | 51'328 | 8'480 | Reference level |  |
| Vegetation low | 4'982 | 5'707 | 0.10 | 0.10 – 0.11 |
| Beach | 227 | 268 | 0.11 | 0.09 – 0.13 |
| Open fields | 28'548 | 67'087 | 0.06 | 0.05 – 0.06 |
| Sea | 255 | 3'531 | 0.01 | 0.01 – 0.01 |
| Hour |  |  | 0.99 | 0.99 – 0.99 |
| **Pogon – Indonesia** |  |  |  |  |
| Habitat |  |  |  |  |
| Roads | 1'176 | 78 | 1.29 | 0.98 – 1.69 |
| Buildings | 2'490 | 225 | Reference level |  |
| Vegetation high | 9'593 | 12'956 | 0.11 | 0.09 – 0.12 |
| Slope |  |  | 0.88 | 0.88 – 0.89 |
| Hour |  |  | 0.98 | 0.97 – 0.98 |
| **Poptún – Guatemala** |  |  |  |  |
| Habitat |  |  |  |  |
| Roads | 9'451 | 5'034 | 1.15 | 1.01 – 1.20 |
| Buildings | 17'491 | 11'033 | Reference level |  |
| Vegetation low | 12'215 | 23'090 | 0.30 | 0.29 – 0.31 |
| Slope |  |  | 1.02 | 1.01 – 1.04 |
| Hour |  |  | 0.99 | 0.99 – 0.99 |
| **La Romana – Guatemala** |  |  |  |  |
| Habitat |  |  |  |  |
| Roads | 1'996 | 186 | 0.32 | 0.26 – 0.39 |
| Buildings | 10'134 | 250 | Reference level |  |
| Vegetation low | 46'316 | 46'662 | 0.04 | 0.03 – 0.04 |
| Vegetation high | 1'385 | 12'733 | 0.01 | 0.00 – 0.01 |
| Slope |  |  | 0.84 | 0.84 – 0.84 |
| Hour |  |  | 0.99 | 0.99 – 0.99 |
| **Sabaneta – Guatemala** |  |  |  |  |
| Habitat |  |  |  |  |
| Roads | 4'553 | 161 | 0.42 | 0.34 – 0.52 |
| Buildings | 19'774 | 258 | Reference level |  |
| Vegetation low | 66'972 | 90'880 | 0.03 | 0.02 – 0.03 |
| Slope |  |  | 0.69 | 0.68 – 0.70 |
| Hour |  |  | 0.99 | 0.99 – 1.00 |

**Supplementary Table 3.** – Results for all study sites with vegetation low resource as reference level in the spatial mixed effect logistic regression model.

|  | **Observed number of observations** | **Randomly generated number of observations** | **Odds ratio (OR)** | **Confidence interval of OR** |
| --- | --- | --- | --- | --- |
| **Habi – Indonesia** |  |  |  |  |
| Habitat |  |  |  |  |
| Roads | 2'560 | 2'827 | 1.21 | 1.13 – 1.30 |
| Buildings | 51'328 | 8'480 | 9.84 | 9.37 – 10.33 |
| Vegetation low | 4'982 | 5'707 | Reference level |  |
| Beach | 227 | 268 | 1.01 | 0.90 – 1.31 |
| Open fields | 28'548 | 67'087 | 0.55 | 0.52 – 0.57 |
| Sea | 255 | 3'531 | 0.09 | 0.08 – 0.11 |
| Hour |  |  | 0.99 | 0.99 – 0.99 |
| **Poptún – Guatemala** |  |  |  |  |
| Habitat |  |  |  |  |
| Roads | 9'451 | 5'034 | 3.78 | 7.12 – 9.74 |
| Buildings | 17'491 | 11'033 | 3.30 | 22.33 – 30.67 |
| Vegetation low | 12'215 | 23'090 | Reference level |  |
| Slope |  |  | 1.02 | 0.14 – 0.16 |
| Hour |  |  | 0.99 | 0.99 – 0.99 |
| **La Romana – Guatemala** |  |  |  |  |
| Habitat |  |  |  |  |
| Roads | 1'996 | 186 | 8.33 |  |
| Buildings | 10'134 | 250 | 26.17 | 2.60 – 3.88 |
| Vegetation low | 46'316 | 46'662 | Reference level |  |
| Vegetation high | 1'385 | 12'733 | 0.15 | 0.02 – 0.02 |
| Slope |  |  | 0.84 | 0.84 – 0.84 |
| Hour |  |  | 0.99 | 0.99 – 0.99 |
| **Sabaneta – Guatemala** |  |  |  |  |
| Habitat |  |  |  |  |
| Roads | 4'553 | 161 | 16.91 | 14.24 – 20.07 |
| Buildings | 19'774 | 258 | 39.81 | 35.13 – 45.12 |
| Vegetation low | 66'972 | 90'880 | Reference level |  |
| Slope |  |  | 0.69 | 0.68 – 0.70 |
| Hour |  |  | 0.99 | 0.99 – 1.00 |

**Supplementary Table 4.** – Results for Habi with the beach as reference level, and for La Romana and Pogon with vegetation high resource as reference levels in the spatial mixed effect logistic regression models for each site.

|  | **Observed number of observations** | **Randomly generated number of observations** | **Odds ratio (OR)** | **Confidence interval of OR** |
| --- | --- | --- | --- | --- |
| **Habi – Indonesia** |  |  |  |  |
| Habitat |  |  |  |  |
| Roads | 2'560 | 2'827 | 1.11 | 0.92 – 1.35 |
| Buildings | 51'328 | 8'480 | 9.05 | 7.53 – 10.87 |
| Vegetation low | 4'982 | 5'707 | 0.92 | 0.76 – 1.11 |
| Beach | 227 | 268 | Reference level |  |
| Open fields | 28'548 | 67'087 | 0.50 | 0.42 – 0.60 |
| Sea | 255 | 3'531 | 0.09 | 0.07 – 0.11 |
| Hour |  |  | 0.99 | 0.99 – 0.99 |
| **Pogon – Indonesia** |  |  |  |  |
| Habitat |  |  |  |  |
| Roads | 1'176 | 78 | 11.98 | 9.44 – 15.20 |
| Buildings | 2'490 | 225 | 9.29 | 8.03 – 10.76 |
| Vegetation high | 9'593 | 12'956 | Reference level |  |
| Slope |  |  | 0.88 | 0.88 – 0.89 |
| Hour |  |  | 0.98 | 0.97 – 0.98 |
| **La Romana – Guatemala** |  |  |  |  |
| Habitat |  |  |  |  |
| Roads | 1'996 | 186 | 55.14 | 46.67 – 65.16 |
| Buildings | 10'134 | 250 | 174.99 | 152.27 – 201.11 |
| Vegetation low | 46'316 | 46'662 | 6.80 | 6.41 – 7.22 |
| Vegetation high | 1'385 | 12'733 | Reference level |  |
| Slope |  |  | 0.84 | 0.84 – 0.84 |
| Hour |  |  | 0.99 | 0.99 – 0.99 |

**Supplementary Table 5.** – Results for Habi with the sea resource as reference levels in the spatial mixed effect logistic regression model.

|  | **Observed number of observations** | **Randomly generated number of observations** | **Odds ratio (OR)** | **Confidence interval of OR** |
| --- | --- | --- | --- | --- |
| **Habi – Indonesia** |  |  |  |  |
| Habitat |  |  |  |  |
| Roads | 2'560 | 2'827 | 13.05 | 11.35 – 15.00 |
| Buildings | 51'328 | 8'480 | 106.06 | 93.07 – 120.87 |
| Vegetation low | 4'982 | 5'707 | 10.78 | 9.43 – 12.33 |
| Beach | 227 | 268 | 11.73 | 9.39 – 14.65 |
| Open fields | 28'548 | 67'087 | 5.90 | 5.18 – 6.81 |
| Sea | 255 | 3'531 | Reference level |  |
| Hour |  |  | 0.99 | 0.99 – 0.99 |


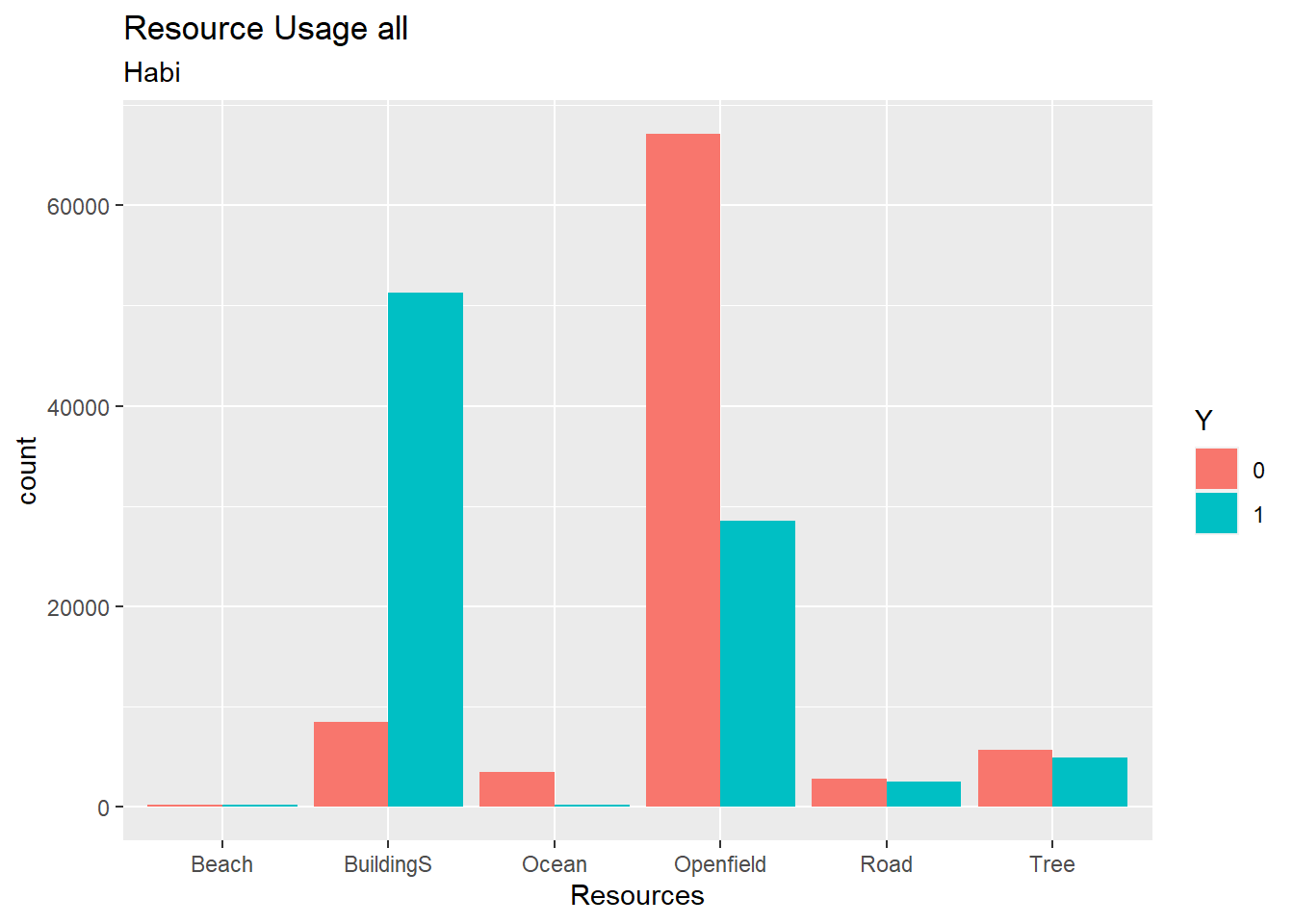


**Supplementary Figure 1.** – Habi's observations resource allocation. Y=0 represents presence of randomly generated fixes while Y=1 represents the presence of GPS fixes in its respective habitat resource.


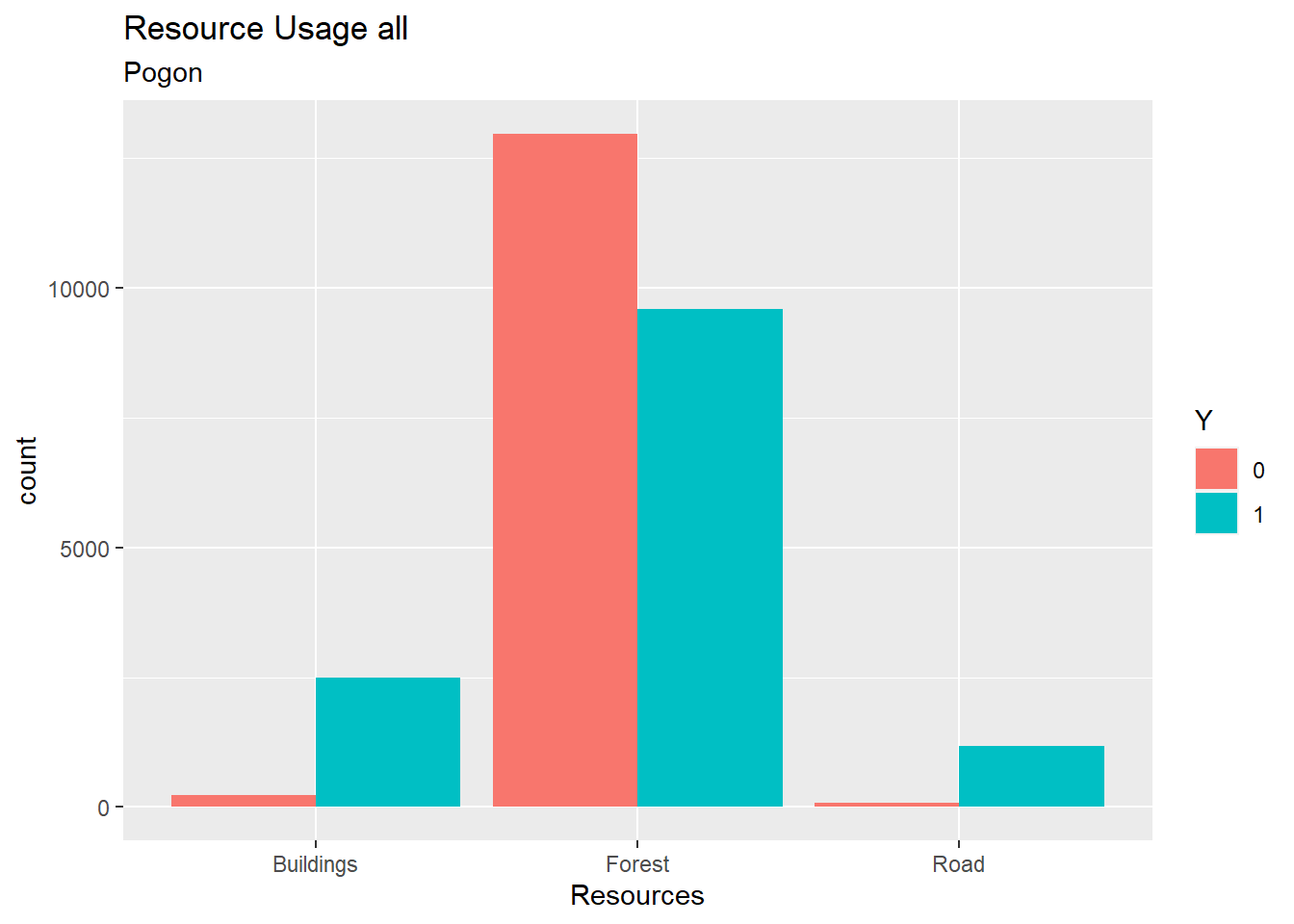


**Supplementary Figure 2.** – Pogon's observations resource allocation. Y=0 represents presence of randomly generated fixes while Y=1 represents the presence of GPS fixes in its respective habitat resource.


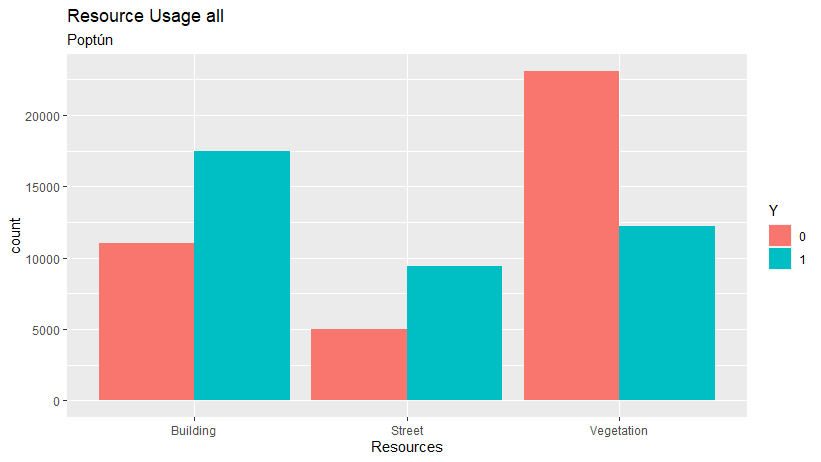


**Supplementary Figure 3.** – Poptún's observations resource allocation. Y=0 represents presence of randomly generated fixes while Y=1 represents the presence of GPS fixes in its respective habitat resource.


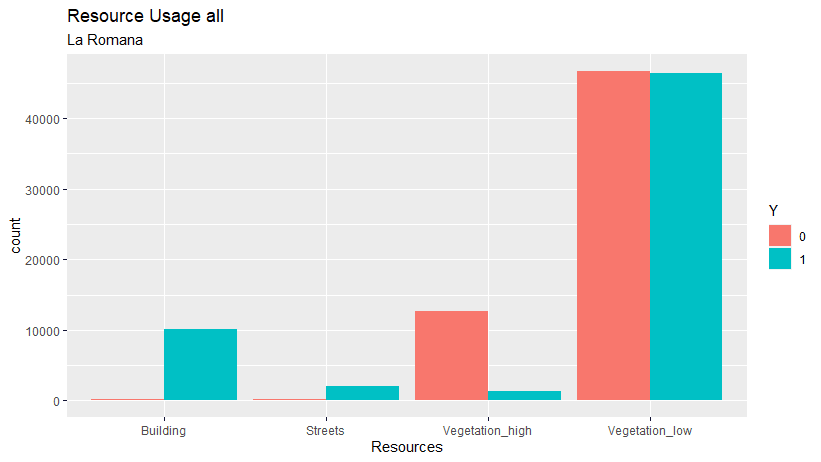


**Supplementary Figure 4.** – La Romana's observations resource allocation. Y=0 represents presence of randomly generated fixes while Y=1 represents the presence of GPS fixes in its respective habitat resource.


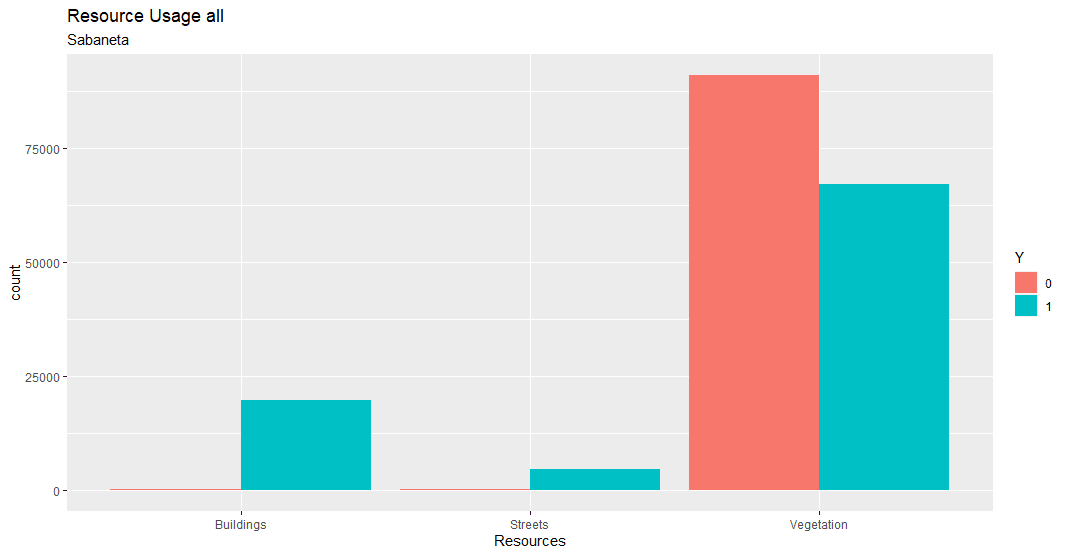


**Supplementary Figure 5.** – Sabaneta's observations resource allocation. Y=0 represents presence of randomly generated fixes while Y=1 represents the presence of GPS fixes in its respective habitat resource.


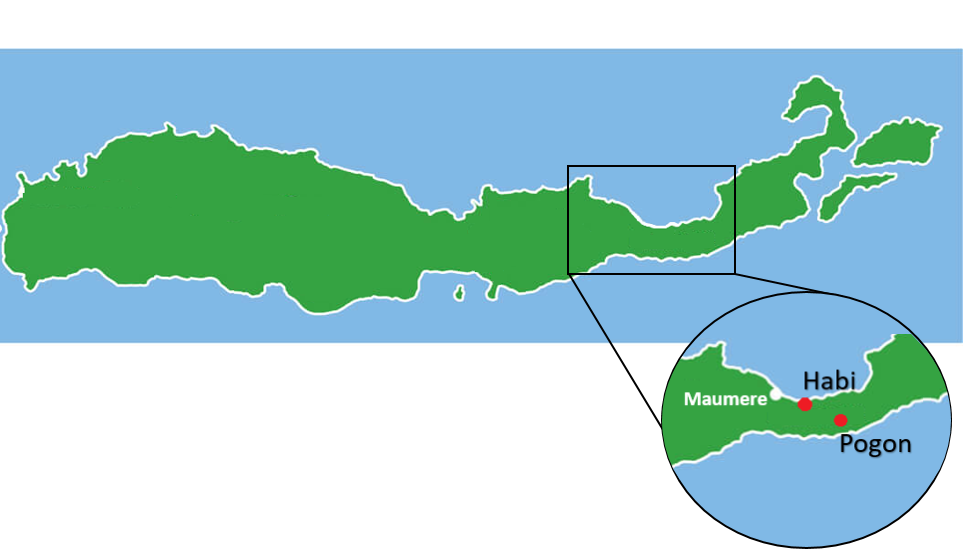


**Scale 1:1,355,651**

**Scale 1:104,612**

**Supplementary Figure 6.** - Localization of the two study areas in Indonesia: Habi and Pogon.


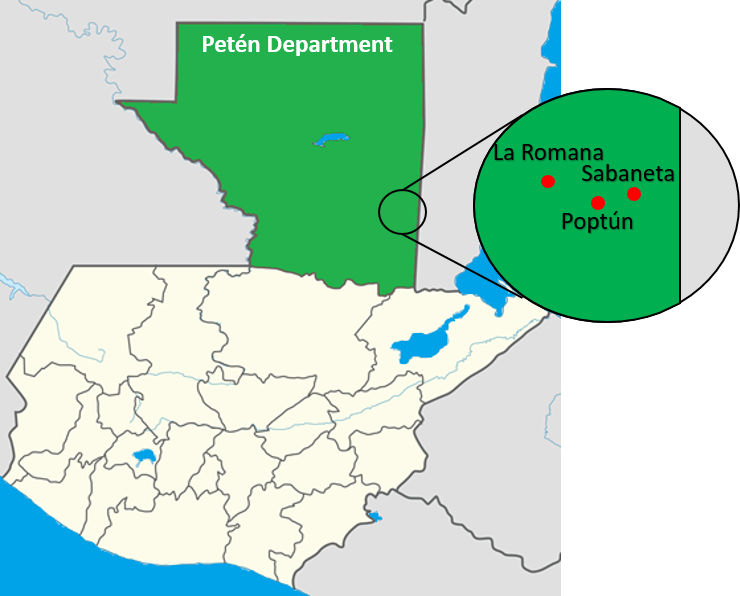


**Scale 1:2,822,359**

**Scale 1:192,842**

**Supplementary Figure 7.** - Localization of the three study areas in Guatemala: Poptún, Romana and Sabaneta.
